# Arthrodèse de la cheville: technique et résultats: à propos de 10 cas

**DOI:** 10.11604/pamj.2014.17.80.3537

**Published:** 2014-01-31

**Authors:** Mohammed El Idrissi, Atif Mechchat, Abdelghani Elayoubi, Mohammed Shimi, Abdelhalim Elibrahimi, Abdelmajid Elmrini

**Affiliations:** 1 Service de Chirurgie Ostéoarticulaire B4, CHU Hassan II, Fès, Maroc

**Keywords:** Cheville, arthrose, arthrodèse, ankle, arthrosis, arthrodesis

## Abstract

L'arthrodèse de la cheville est le traitement de choix dans les atteintes articulaires importantes de la cheville afin de lui restituer une indolence parfaite et une bonne stabilité. L'objectif de notre travail est d’évaluer les résultats à moyen terme de l'arthrodèse de la cheville pour arthrose post-traumatique à travers L’étude d'une série de dix patients. Nous avons menu une étude rétrospective étalée entre janvier 2009 et juin 2013, portant sur dix cas d'arthrose post-traumatique de la cheville traités par arthrodèse. Selon la technique de Méary, utilisant une voie d'abord antérolatérale de la cheville avec une fixation par deux vis. L’âge moyen au moment du traitement est de 45 ans. La consolidation a été obtenue dans un délai moyen de 10 semaines. Au recul moyen de 30 mois, sur les dix patients 7 se sont estimés satisfaits, 2 moyennement satisfait et un patient non satisfait vu la persistance de douleurs. Le score moyen de L'AOFAS postopératoire est de 69,6. L’étude de notre série, nous a permis de confirmer l'intérêt l'arthrodèse dans le traitement de l'arthrose post-traumatique de la cheville en assurant à la fois indolence et stabilité.

## Introduction

L'arthrose primitive de la cheville est une pathologie exceptionnelle en regard de la fréquence de L'arthrose post-traumatique. Les séquelles des fractures bimalléolaires représentent de loin l’étiologie la plus fréquente en raison de la prévalence de ces lésions qui occupent le troisième rang des traumatismes des membres après les fractures de L'extrémité inférieure du radius et celles de L'extrémité supérieure du fémur [1]. Les possibilités thérapeutiques sont multiples, allant du traitement conservateur purement symptomatique au traitement chirurgical, à savoir L'arthrodèse de la cheville ou, depuis quelques années, la mise en place d'une prothèse totale. Le traitement par l′arthrodèse donne de bons résultats avec une grande fiabilité, la prothèse conserve une mobilité mais avec des reculs encore modestes [2]. Le but de notre travail est d’évaluer les résultats à moyen terme de L'arthrodèse de la cheville pour arthrose post-traumatique à travers L’étude d'une série de dix patients.

## Méthodes

Il s'agit d'une étude rétrospective étalée entre janvier 2009 et juin 2013, portant sur dix cas d'arthrose post-traumatique de la cheville traités par arthrodèse. Nous avons inclus dans cette étude tout patient présentant une arthrose post-traumatique de la cheville traitée par arthrodèse, et nous avons exclus toute arthrose d'une autre origine, ou arthrodèse pour une autre indication. Le Tableau 1 représente les traumatismes à L'origine de L'arthrose

**Tableau 1:** Traumatismes à l'origine de l'arthrose

Traumatisme	Nombre de patients
Fractures bimalléolaires	**6**
Fracture pilon tibial	**3**
Fracture astragale	**1**

**Figure 1:**
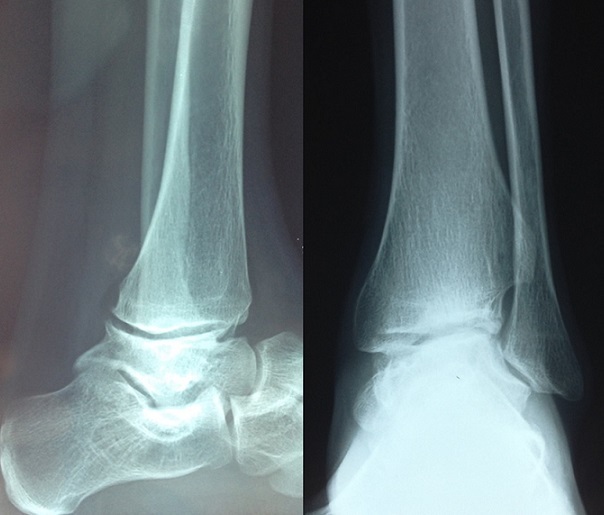
Cliché préopératoire montrant une arthrose de la cheville suite à une de nécrose post-traumatique du talus

**Figure 2:**
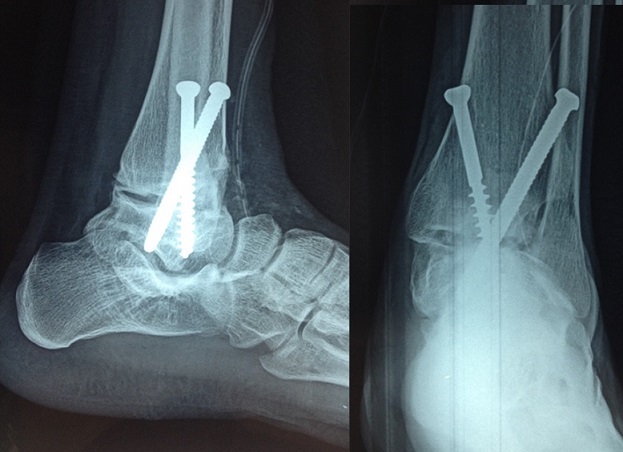
Contrôle postopératoire après arthrodèse

La technique chirurgicale que nous avons adopté est celle décrite par Méary, utilisant une voie d'abord antérolatérale de la cheville [3]. Le pied est fixé dans le plan horizontal entre 0 et 5°, dans le plan frontal entre 0 et 5° de valgus de L'arrière-pied et dans le plan sagittal à 90° de flexion dorsale. La fixation est réalisée par deux vis grand fragment croisées dans les trois plans de L'espace. L'immobilisation postopératoire est assurée par une attelle puis une botte plâtrée maintenu pendant 8 semaines. La rééducation est entamée dés ablation du plâtre et elle vise essentiellement les articulations de L'avant pied (Figure 1, Figure 2)

L’évaluation postopératoire a été basée sur les éléments cliniques notamment la stabilité de la cheville à L'examen clinique, et également la présence ou non de douleurs. Les clichés radiologiques de face et de profile ont permis d ‘apprécier la consolidation par L'apparition de ponts osseux trabéculaires couvrant au moins 50% de L'espace tibiotalien, ainsi que l'alignement de la cheville. L’évaluation subjective a été appréciée en précisant le degré de satisfaction des patients: en satisfait, moyennement satisfait ou non satisfait. L’évaluation objective a été basée sur le score de L'AOFAS (American Orthopedic Foot and Ankle Score) [4].

## Résultats

Nous avons opérés dix patients. L’âge moyen au moment du traitement est de 45 ans (±8) (36-61). Ce groupe de patient comporte 6 hommes et 4 femmes. Le recul moyen est de 30 mois (±9) (18-48). La consolidation a été obtenu dans un délai moyen de 10 semaines (±2,84 (8-18)). Nous n'avons noté aucun cas d'infection postopératoire, aucune complication thérapeutique liée à la procédure, ni de pseudarthrodèse. Chez deux patients nous avons noté la persistance d'un valgus (de 5 et 6°). Un patient a développé une algodystrophie. Nous avons noté également deux cas d'arthrose secondaire. Au recul moyen, sur les dix patients 7 se sont estimés satisfaits, 2 moyennement satisfait et un patient non satisfait vu la persistance de douleurs. Le score moyen de L'AOFAS postopératoire est de 69,6 (± 15.09) (45 - 90).

## Discussion

La cheville est une articulation complexe subissant une force tridimensionnelle: verticale de compression, tangentielle antéropostérieure, latéromédiale de cisaillement et de rotation [1]. L'arthrose de la cheville est dans plus de 80% des cas d'origine post-traumatique et devient symptomatique et gênante dans la vie de tous les jours pour le patient en règle générale de nombreuses années après le traumatisme initial [2, 5]. Cette arthrose se manifeste essentiellement par des douleurs avec un retentissement fonctionnel major [6]. Le traitement d'une telle affection de L'appareil locomoteur a suscité beaucoup de discussion, ainsi deux attitudes s'opposent: le traitement par prothèse qui est devenu plus populaire et qui conserve une mobilité mais avec des reculs encore modestes et le traitement par arthrodèse qui donne de bons résultats avec une grande fiabilité. Celle-ci reste le traitement de choix dans les atteintes articulaires importantes de la cheville afin de lui restituer une indolence parfaite et une bonne stabilité [7]. La première arthrodèse de la cheville a été réalisée en 1879 par Albert qui a réalisé pour la première fois une ankylose chirurgicale de la cheville à laquelle il a donné le nom d'arthrodèse, et depuis environ 40 procédures ouvertes ont été décrites [1, 8]. Ces techniques peuvent être regroupées en deux groupes: la stabilisation par voie interne (vissage, plaque antérieure, greffons osseux) ou la stabilisation par voie externe utilisant un fixateur comme moyen de compression de L'articulation. Le concept de Charnley comporte une technique basée sur un débridement associé à une fixation externe mais la stabilité est obtenue dans un seul plan [9]. Plusieurs modifications ont été rapportées sur la technique de fixation externe pour en améliorer les résultats [10, 11]. Saragaglia et al. ont rapporté les résultats de 18 arthrodèses utilisant une fixation externe avec comblement de L'espace de résection par les greffons spongieux Le taux de fusion rapporté est de 100% dans un délai de 3 à 6 mois. Aucune complication majeure n'est à déplorer [12]. La technique de Méary a été décrite par lui-même puis fut largement diffusée [3, 13]. Elle consiste en un abord antéro-externe qui donne un excellent jour sur la face antérieure de l′articulation, la fixation fait appel à deux vis croisées dans tous les plans et la reconstitution d′un plan aponévrotique continu isolant le plan ostéoarticulaire du plan cutané [14]. L'arthrodèse arthroscopique a été au début utilisée par Schneider en 1983, puis elle est devenue une alternative fiable aux arthrodèses à foyer ouvert [15–18]. Cependant la sélection des patients est un facteur essentiel influant les résultats fonctionnels; L'arthroscopie est utile principalement pour l ‘arthrodèse in situ, la nécessité de correction de déformations en varus ou valgus ou en rotation constitue une limite à L'arthroscopie et nécessite un abord direct pour d’éventuelles ostéotomies [8].

Les résultats fonctionnels que nous avons pu obtenir sont si satisfaisants. Mognon [7] utilisait une fixation par haubanage le score AOFAS moyen était de 71, 1. Chez Haddad et al. [19] qui ont publié les résultats 49 séries de la littérature ce score était de 75,6 et chez Kein et al. [20]qui ont utilisé une fixation externe il était à 69,3. Dans notre série nous avons noté un cas de retard de consolidation (18 semaines) mais, pas de pseudarthrose, ceci s'explique peut être par le nombre réduit de patient inclus dans cette étude. Si la principale complication liée à la technique de Méary est la nécrose cutanée, la manipulation douce et la fermeture du plan aponévrotique nous ont permis d’éviter une telle complication chez nos patients.

## Conclusion

L'arthrodèse de la cheville est un moyen thérapeutique simple et efficace, très utile pour le traitement de L'arthrose post-traumatique de la cheville. Certes elle condamne une articulation si primordiale au bon fonctionnement du membre inférieur au cours de la marche, mais elle permet de soulager le patient d'une souffrance si onéreuse surtout de la douleur. La comparaison avec L'arthroplastie totale de la cheville fait toujours objet de discussion, mais nous pensons que c'est un moyen très rentable surtout dans les pays en voie de développement où les moyens font défaut.
